# mTOR inhibitor Everolimus-induced apoptosis in melanoma cells

**DOI:** 10.1007/s12079-019-00510-0

**Published:** 2019-03-08

**Authors:** Dorota Ciołczyk-Wierzbicka, Marta Zarzycka, Dorota Gil, Piotr Laidler

**Affiliations:** Medical Biochemistry, Jagiellonian University Medical College, ul. Kopernika 7, 31-034 Kraków, Poland

**Keywords:** Melanoma, Apoptosis, Caspase-3 activity, Proliferation, Protein kinase inhibitors, mTOR

## Abstract

Melanoma is the most aggressive, therapy-resistant skin cancer. The mammalian target of rapamycin (mTOR), the serine/threonine kinase which integrates both intracellular and extracellular signals, plays a crucial role in coordinating the balance between the growth and death of cells. The object of this study is a comparison of the influence of mTOR inhibitor everolimus in the concentration range between 20 nM and 10 μM, used individually and in combination with selected downstream protein kinases inhibitors: LY294002 (PI3K), U0126 (ERK1/2), AS-703026 (MEK) and MK-2206 (AKT) on the expression of pro-survival proteins: p-Bcl-2 (S70), p-Bcl-2 (T56), Bcl-2, Bcl-xL, Mcl-1, activity of caspase-3, proliferation and induction of apoptosis in melanoma cells. Current results clearly show that the nanomolar concentration of the mTOR inhibitor everolimus in combination with the inhibitor of MAP kinase (AS-703026) or AKT kinase (MK-2206) is effective in inducing apoptosis and reducing proliferation of melanoma cells. The herein research results confirm the hypothesis on the important role of mTOR signaling in cancer progression, and gives hope that implementation of successful combination of its inhibitors will find recognition and application in cancer treatment in the near future.

## Introduction

Apoptosis, or programmed cell death, plays an important role in controlling number of cells in many developmental and physiological processes and in oncotherapy-induced killing of cancer cells (Galluzzi et al. 2018). It is a structured, genetically regulated biological process guided by the ratio of pro-apoptotic and anti-apoptotic proteins (Hu and Kavanagh 2003).

In particular anticancer therapies, it is important to understand the mechanisms associated with cell death as it is believed that besides inhibition of tumour growth and cell invasion, the effectiveness of anticancer therapy depends mainly on its ability to induce apoptosis in cancer cells (Pfeffer and Singh 2018).

The mTOR protein is a serine/threonine protein kinase consisting of two complexes: mTORC1 and mTORC2. The mTORC1 complex activates two best characterized downstream effectors: S6 ribosomal kinase1 (S6K1) and eukaryotic initiation factor 4E-binding protein 1 (4E-BP1), and initiates translation of key proteins for regulation of metabolism and processes that are fundamental to cell growth, proliferation, cell cycle and autophagy (Watanabe et al. 2011; Paquette et al. 2018). It seems that the basic function of the TORC2 complex is cytoskeletal organization and regulation of cell survival and invasion (Kim et al. 2017).

Dysregulation or stimulation of PI3K-AKT and mTOR pathway plays a significant role in oncogenesis (Yang et al. 2017; Li et al. 2018). Overexpression of proteins of this pathway, and intensified intracellular signal transduction have been confirmed in numerous types of cancer including breast, ovarian, prostate, gastric, kidney, bladder, melanoma, hepatocellular carcinoma (Kim et al. 2017; Ruzzolini et al. 2017; Conciatori et al. 2018), and tumours of hematological origin, such as acute leukemia, mantle cell lymphoma, Hodgkin’s disease or multiple myeloma (Barrett et al. 2012).

Large-scale randomized trials have demonstrated that everolimus prolongs survival of patients with solid cancers, such as advanced breast cancer, renal cell carcinoma, and several kinds of neuroendocrine tumour (Lin et al. 2016; Kim et al. 2017; Li et al. 2018). Literature data Weeber et al. (2017) also suggest that the benefits of everolimus-based therapy depend on the genetic status of mutations in B-RAF and Phosphatase and Tensin Homolog (PTEN). The loss of function or aberration of PTEN is associated with the success of treatment, while B-RAF wild type could be responsible for the resistance. PTEN status may potentially affect the choice of clinical treatment and require reduced agent doses, thereby reducing toxicity in combined inhibition of the MEK/ERK, PI3K/AKT and mTOR pathways (Sathe et al. 2018). Limited anti-tumour effects of mTOR inhibitors (rapalogs), may be related to the induction of signaling feedback loops (Conciatori et al. 2018; Sathe et al. 2018]. In view of the above, the simultaneous blocking of both signaling pathways – PI3K/AKT and mTOR – can be an effective therapeutic strategy on account of promoting prolonged AKT, S6K1 and 4E-BP1 dephosphorylation and induction of apoptosis (Conciatori et al. 2018; Sathe and Nawroth 2018).

Literature data (Kim et al. 2017) and our own results (Ciołczyk-Wierzbicka and Laidler 2018; Ciołczyk-Wierzbicka et al. 2018) suggest that mTOR inhibitors – both rapamycin and everolimus – have significant impact on cell cycle regulation, reduction of cell proliferation and invasiveness of melanoma cells (Ciołczyk-Wierzbicka and Laidler 2018; Ciołczyk-Wierzbicka et al. 2018). They also inhibit expression of anti-apoptotic protein as well as induce apoptosis and autophagy (Kim et al. 2017).

Since many current studies searching for effective anticancer treatment focus their efforts on new capabilities of the already registered drugs and their use in combination with other potential ones, the herein study focuses on the effects of protein kinase inhibitors, and in particular the very promising mTOR inhibitor everolimus, on expression of pro-survival Bcl-2 family proteins, caspase-3 activity, and induction of apoptosis in melanoma cells.

## Materials and methods

### Cell culture

Human melanoma cell lines: primary WM115 (VGP) and metastatic: WM266–4 (derived from metastatic site – right thigh skin). These cells lines feature the specific V600D mutation in the B-RAF gene, as well as express PTEN loss of function including hemizygous deletion and wild type for N-ras, c-KIT and CDK4. Cells were cultured in RPMI-1640 medium supplemented with 10% fetal bovine serum and antibiotics: penicillin and streptomycin. Cells were incubated at 37 °C in a humidified atmosphere of 5% CO_2_ in air. Cells were treated with inhibitors of: 1/AKT – MK-2206 (Selleck) at 2 μM concentration, 2/ MEK – AS-703026 (Selleck) at 10 μM concentration, 3/ PI3K – LY294002 (Cell Signalling TM) at 20 μM concentration, 4/ ERK1/2 – U0126 (Cell Signalling TM) at 10 μM concentration, and 5/ mTOR – everolimus (Selleck) at: 20 nM, 2 μM, 5 μM and 10 μM concentrations. The incubation time of melanoma cells with inhibitors were 24 and 48 h. Cells were obtained from the ESTDAB Melanoma Cell Bank (Tubingen, Germany).

### Caspase-3 activation assay

Activation of caspase-3 in response to applied inhibitors was estimated by means of fluorogenic substrate DEVD-AFC (Biovision). The cells were seeded in 24-well plates (5*10^4^cell/well). After 24 h growth period, they were treated with individual selected inhibitors or their combinations, as well as with vehicle (DMSO) as control. After respective incubation time, the cells were lysed in lysis buffer (50-mM Tris-HCl, pH 7.6, 100-mM NaCl, 1-mM EDTA, 1% Triton X-100) and incubated (1,5 h; RT) with fluorogenic substrate (20 mmol/l Ac-DEVD-AFC). To stop the reaction, the inhibitor of caspase-3 activity (200 nM/l DEVD-CHO) was added, and the amount of AFC released during incubation was measured by means of spectrofluorometer (Hitachi-2000; λex = 400 nm and λem = 505 nm). The signal corresponding to the caspase-3 activity was normalized to the cell number (determined using the violet crystal test) and reported as fold increase of DEVD-like caspase-3 activity in relation to control sample.

### DNA fragmentation ELISA assay

Apoptosis induction was verified by assessment of the intracellular level of cytoplasmic histone-associated-DNA-fragments – Cell Death Detection ELISA Plus (11,774,425,001 ROCHE) according to the manufacturer’s protocol.

The cells were briefly seeded on 96 wells plate, grown for 24 h and then treated with selected inhibitors, either individual or in combinations, as well as with vehicle (DMSO) as the control. After the incubation period, the cell culture medium was discarded, the cells were lysed, and the lysates were centrifuged in order to eliminate intact cell nuclei. Next, the supernatant with histone-linked DNA fragments was applied on streptavidin-coated microplate well, incubated with immunoreagent, and then treated with a substrate to receive colorimetric signal (A405; BIO-TEK Synergy HT Plate Reader). The results are presented as mean absorbance value (405 nm) which reflects the amount of mono- and oligonucleosomes released to the cytoplasm of apoptotic cells. Furthermore, as per the manufacturer’s instruction, the enrichment factor (EF) was calculated to estimate the fold increase of DNA fragmentation in the treated samples with reference to the control one.

### Cell proliferation

The proliferation of cells was assessed with the crystal violet test as previously described (Ciołczyk-Wierzbicka et al. 2012).

### Cytotoxicity assay

Cytotoxicity of selected kinases’ inhibitors: AKT – MK-2206 (2 μM), MEK – AS-703026 (10 μM), PI3K – LY294002 (20 μM), ERK1/2 – U0126 (10 μM), mTOR – everolimus (20 nM, 2 μM, 5 μM and 10 μM) was determined using Cytotoxicity Detection Kit LDH (Roche, Germany).

### Western blot analysis

Samples for SDS-PAGE electrophoresis were prepared in the same way as previously described (Ciołczyk-Wierzbicka et al. 2012). Antibodies against Phospho-Bcl-2 (Ser70) (SH2) #2827, Phospho-Bcl-2 (Thr56) #2875, Bcl-2 (D55G8) 4223#, Bcl-xl (54H6) 2764#, Mcl-1 (D35A5) 5453# (Cell Signaling Technology), and β-actin (A2228, SIGMA) were used to detect the indicated proteins. Bands were visualized with the use of horseradish peroxidase-coupled secondary anti-mouse or anti-rabbit antibody (Cell Signaling Technology). Immunoreactivity of protein was detected with the use of chemiluminescence, and images were captured with a ChemiDoc MP Imaging System (Bio-Rad Labs). To obtain quantitative results, immunoblots were scanned with the use of SynGene Gene Tools version 4.03.0 (Synoptics Ltd. Beacon House, Nuffield Road Cambridge, CB4 1TF, UK). Densitometry was performed to normalize to β-actin protein level. Presented are representative membranes of at least three independent experiments.

### Densitometry analysis

Densitometry of western blot analysis was performed with the use of SynGene Gene Tools version 4.03.0 (Synoptics Ltd. Beacon House, Nuffield Road Cambridge, CB4 1TF, UK) and the results were normalized to β-actin protein level. Presented are representative membranes of at least three independent experiments with similar results.

### Statistics

Statistical analyses were performed using one-way ANOVA with post-hoc Dunett test (Statistica 12.0 StatSoft). The statistical significance is presented in Figures.

## Results

### Cytotoxicity assay

In all examined melanoma cell lines, inhibitors:: AKT – MK-2206 (2 μM), MEK – AS-703026 (10 μM), PI3K – LY294002 (20 μM), ERK1/2 – U0126 (10 μM), mTOR – everolimus (20 nM, 2 μM, 5 μM and 10 μM) showed no cytotoxicity effect in the 24 h of treatment. LDH activity in the culture medium in no case exceeded 3,8%; however, after 72 h treatment, the effect of cytotoxicity was noticed for combinations of inhibitors: AS-703026 with LY294002 (13%), and MK-2206 with U126 (11%). Increased lactate dehydrogenase activity for the WM266–4 cell line for combination of inhibitors: AS-703026 and MK-2206 (7,1%) was also observed.

### Expression Bcl-2 family pro-survival protein

**Fig. 1:**
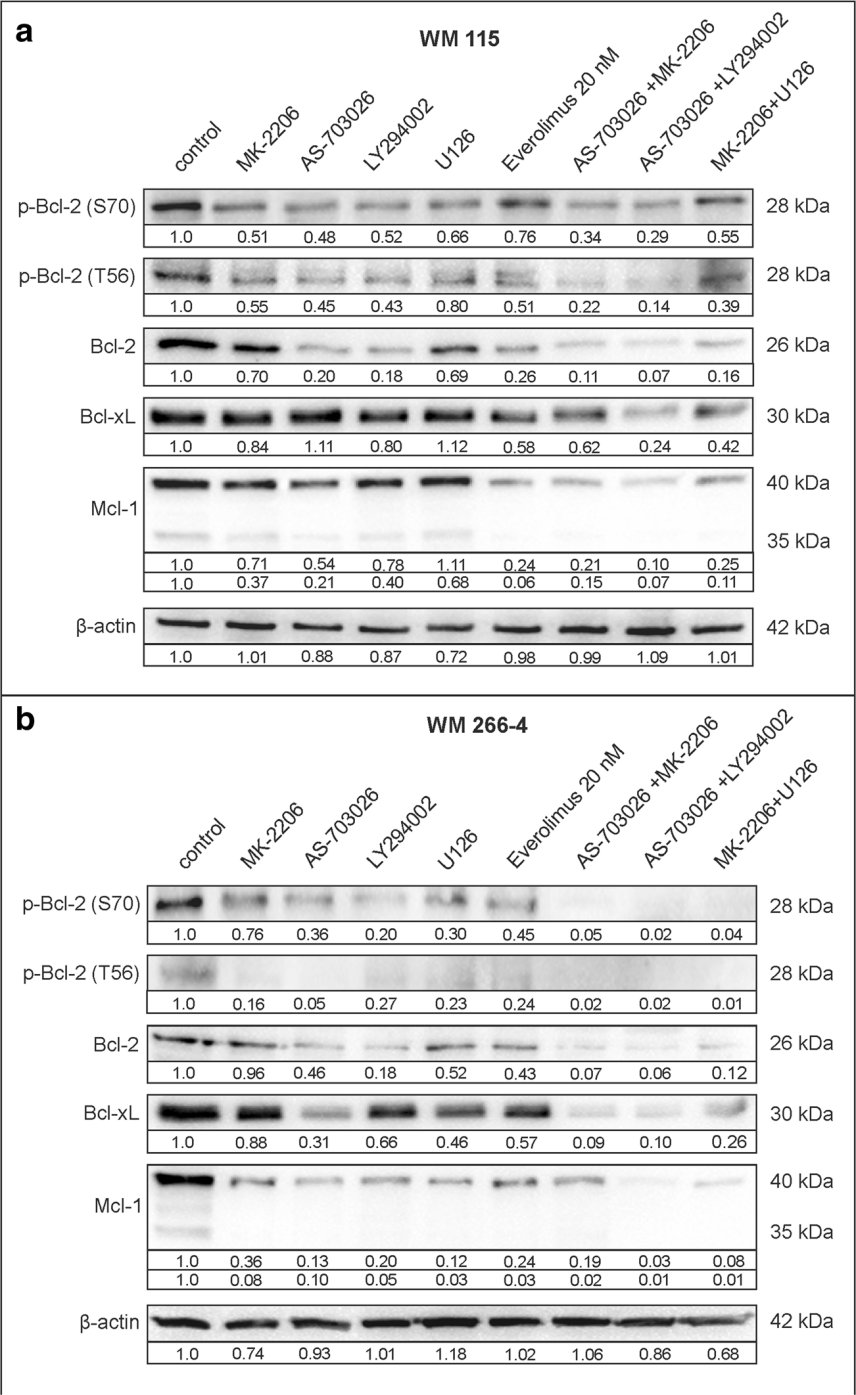
Expression of pro-survival proteins in: WM115 (**a**) and-WM266–4 (**b**). Western blot analysis was performed in accordance with Materials and Methods. Densitometry analyses of western blot were performed on raw volume (sum of intensities of bound-volume calculated from the area of the peak) using SynGene Gene Tools version 4.03.0 (Synoptics Ltd. Beacon House, Nuffield Road Cambridge, CB4 1TF, UK). Densitometry results were normalized to control (melanoma cells untreated with protein kinase inhibitors). Presented are representative of at least three independent experiments

The current study focuses on the comparison of the influence of mTOR inhibitor everolimus, and proteins kinase inhibitors: AKT - MK-2206, PI3K - LY294002 and ERK1/2 - U126 and their combinations on the expression of pro-survival proteins: p-Bcl-2 (S70), p-Bcl-2 (T56), Bcl-2, Bcl-xL, and Mcl-1 in metastatic (WM 266–4) and primary (WM 115) melanoma cell lines (Fig. 1).

The protein kinase inhibitors, when used individually, produced a decrease in the expression of pro-survival proteins, whereas, their combinations had significantly more pronounced effects (Fig. 1).

The most visible effect of melanoma cells treatment with single inhibitor on the protein expression level of p-Bcl-2 (S70) and p-Bcl-2 (T56) was observed after incubation with MEK inhibitor - AS-703026 or PI3K inhibitor - LY294002, slightly weaker being that for ERK1/2 inhibitor - U126 or AKT - MK-2206 (Fig.1). The expression level of total Bcl-2 also decreased in WM 266–4 and WM 115 cell lines. Additionally, AS-703026 inhibitor significantly reduced the expression level of Bcl-xL (by about 70%) in WM 266–4 (Fig. 1b).

Moreover, the combinations of inhibitors significantly affected the expression level of analysed pro-survival proteins. The most significant, nearly 100% reduction, was observed in WM 266–4, and 80% decrease in WM 115 after their treatment with combination of MEK inhibitor -AS-703026 and PI3K - LY294002 (Fig.1). Slightly worse results were obtained for the combination of inhibitors: AS-703026 with MK-2206, and MK-2206 with U126 in both melanoma cell lines (Fig. 1).

**Fig. 2:**
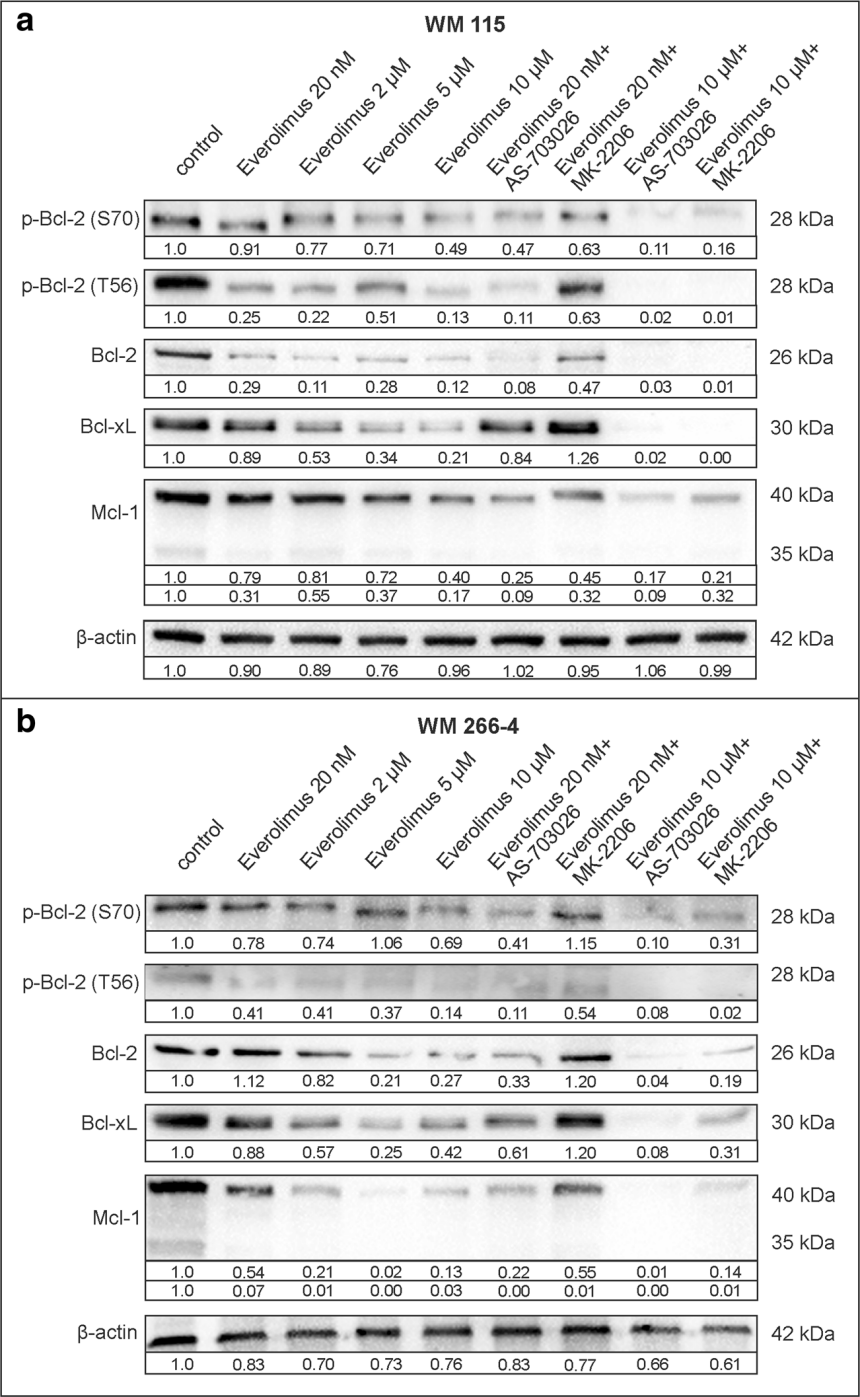
The effect of treatment of melanoma cells with everolimus on expression of pro-survival Bcl-2 family protein. Western blot analysis of: WM115 (**a**) and-WM266–4 (**b**) melanoma cell lines. Densitometry analyses of western blot were performed on raw volume (sum of intensities of bound-volume calculated from the area of the peak) using SynGene Gene Tools version 4.03.0 (Synoptics Ltd. Beacon House, Nuffield Road Cambridge, CB4 1TF, UK). Densitometry results were normalized to control (melanoma cells untreated with protein kinase inhibitors). Presented are representative of at least three independent experiments

The effect of different doses of everolimus (20 nM, 2 μM, 5 μM, and 10 μM) as well as the combination of everolimus (20 nM and 10 μM) with AS-703026 and MK-2206 on the expression of pro-survival proteins was also evaluated. The most visible reduction of the expression level of studied proteins was observed after treatment of WM115 and WM266–4 with 10 μM everolimus. (Fig. 2). However, its 5 μM concentration reduced Mcl-1 expression by almost 100% in WM 266–4. Addition of AS-703026 to 10 μM everolimus contributed to the most significant reduction of apoptotic proteins expression levels in both melanoma cell lines where all evaluated proteins were reduced by more than 80%. This effect was also evident at 20 nM everolimus concentration (Fig. 2). Somewhat surprisingly, a slight increase in the expression of Bcl-2, Bcl-2 (S70) and Bcl-xL in WM266–4 using a combination of 20 nM everolimus and MK-2206 was noted. The use of AKT inhibitor – MK2206 in combination with an mTOR inhibitor everolimus was less effective; however, the clear-cut effect was observed at everolimus 10 μM concentration, the highest level used (Fig. 2).

### Caspase-3 activity and cell proliferation in melanoma cell lines

Investigated was also the effect of protein kinases’ inhibitors: AKT – MK-2206, MEK – AS-703026, PI3K – LY294002 and ERK1/2 – U126 and mTOR – everolimus in a single mode, and their combinations on caspase-3 activation and proliferation in WM266–4 and WM115 melanoma cell lines.

**Fig. 3:**
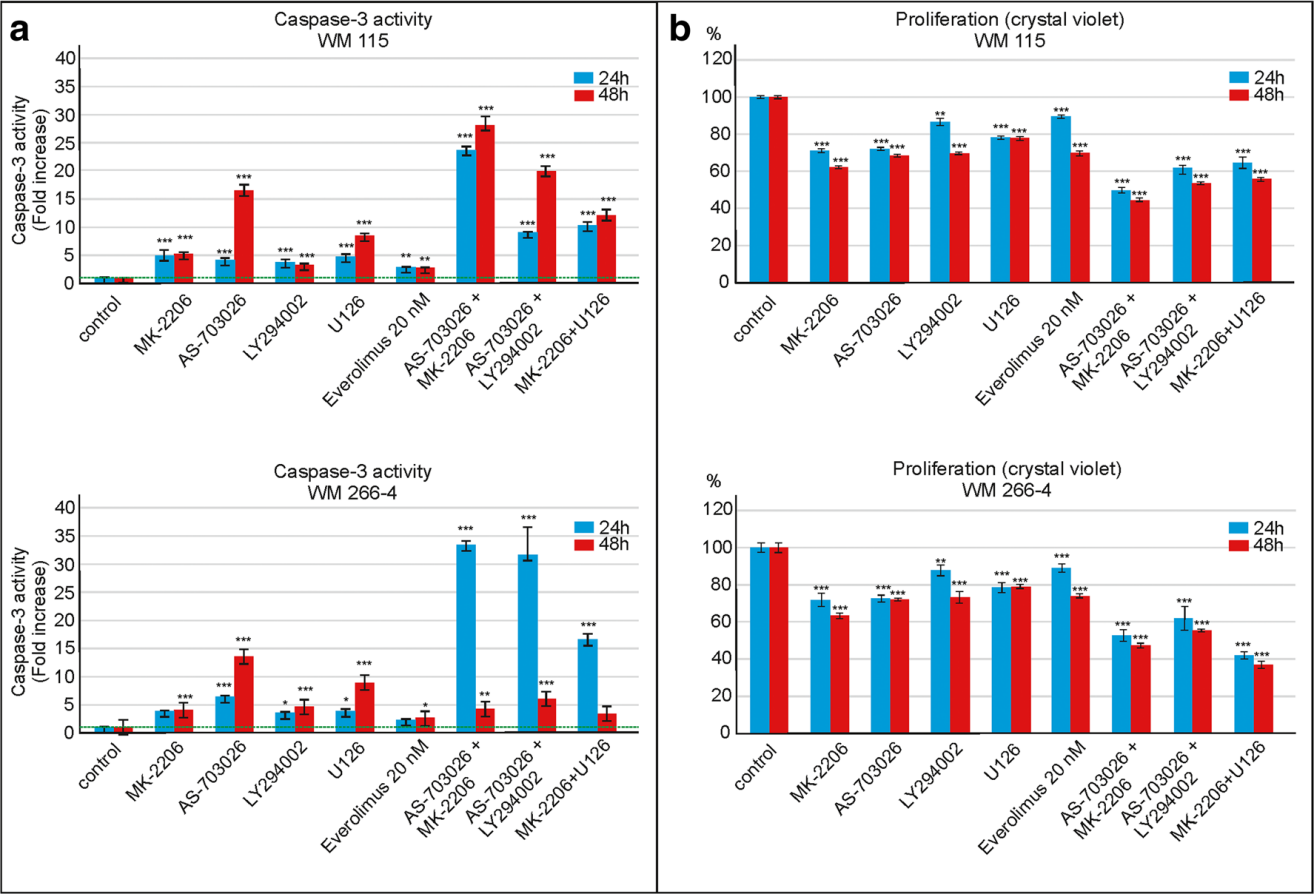
The effect of protein kinase inhibitors on caspase-3 activity and cell proliferation in WM115 and WM266–4 melanoma cell lines. The caspase-3 activity (**a**) and cell proliferation - crystal violet assay (**b**) were calculated from mean values of three independent experiments. Statistical analyses were performed using one-way ANOVA with post-hoc Dunett test (Statistica 12.0 StatSoft); significant difference: (*) *p* < 0.05, (**) *p* < 0.01, (***) *p* < 0.001

The highest increases of caspase-3 activity in the case of individually-used protein kinase inhibitors were observed for both cell lines after application of MEK kinase inhibitor – AS-703026. A weaker yet meaningful effect occurred for ERK1/2 inhibitor - U126. A few-fold, although not particularly significant, increase of caspase-3 activity was also observed for AKT inhibitor – MK-2206 and PI3K - LY294002 (Fig. 3a). For both melanoma cell lines, treatment with protein kinase inhibitors was accompanied by a decrease in proliferation: highest after the use of the AKT inhibitor - MK2206 (about 40%), and slightly smaller for inhibitors of the MAP kinase pathway, AS-703026 or U126 (Fig. 3b).

The combination of inhibitors was accompanied by substantially higher increases of caspase-3 activity. The combination of MEK inhibitor – AS-703026 and AKT inhibitor – MK-2206 led to up to 35-fold increase of caspase-3 activity (*p* < 0.001), whereas somewhat lower, 15-fold (*p* < 0.001), was observed for the combination of the MEK inhibitor - AS-703026 and PI3K inhibitor - LY294002 (*p* < 0.001). Still a high, but much lower increase was noticed for the combination of inhibitors: ERK1/2 - U126 with AKT- MK-2206 (*p* < 0.001) (Fig. 3a). 48 h treatment yielded a higher increase of caspase-3 activity in comparison to 24 h treatment in each case of inhibition, except for the use of the combination of inhibitors in line WM266–4, suggesting faster caspase-3 activation in this cell line (Fig. 3a).

In the case of proliferation, the greatest decrease was observed for the combination of inhibitors: MK-2206 with U126 for WM266–4 cell line (about 63%), and AS-703026 with MK-2206 for WM115 cell line (about 55%) (Fig. 3b).

**Fig. 4:**
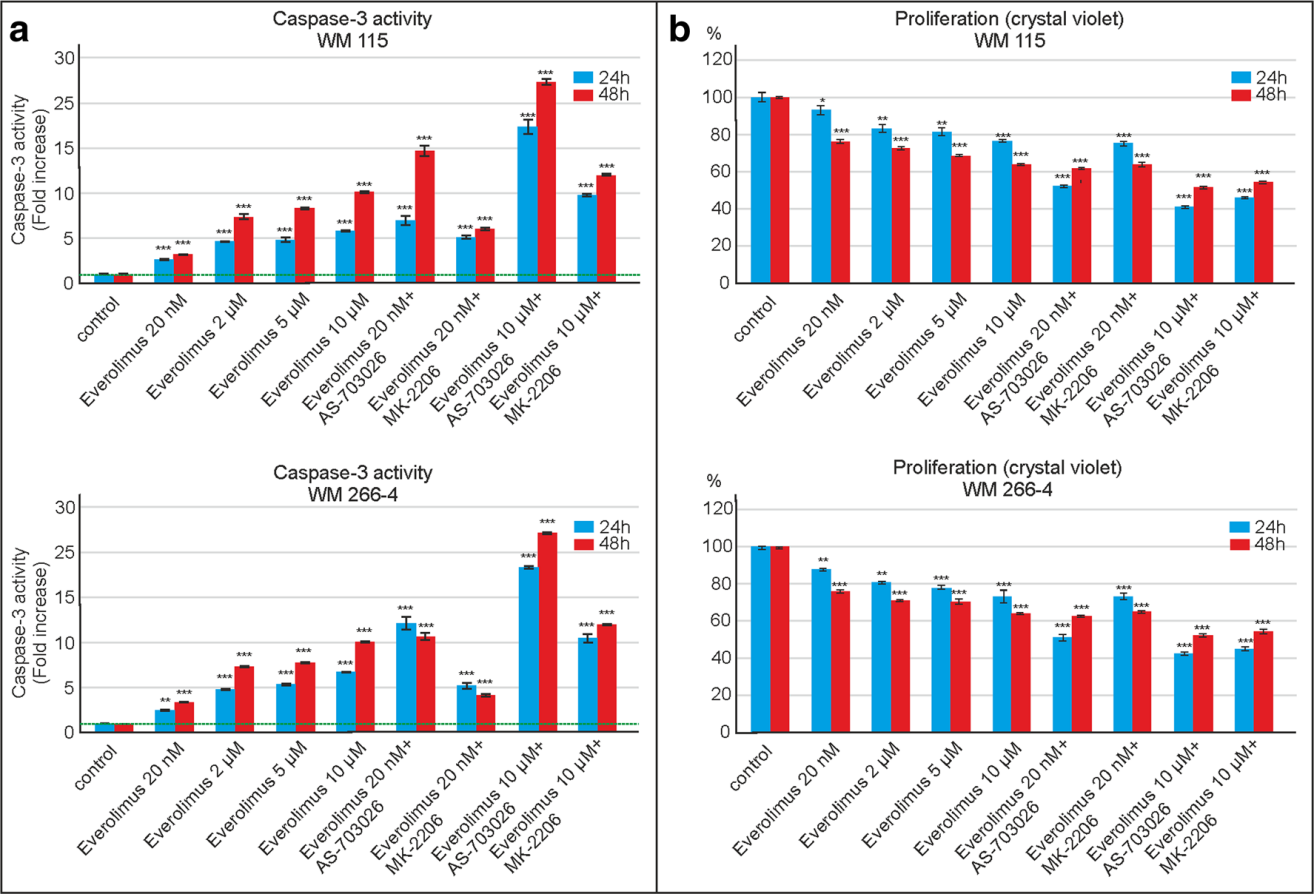
The effect of everolimus on caspase-3 activity and cell proliferation in: WM115 and WM266–4 melanoma cell lines. The effect of caspase-3 activity (**a**) and cell proliferation - crystal violet assay (**b**) were calculated from mean values of three independent experiments. Statistical analyses were performed using one-way ANOVA with post-hoc Dunett test (Statistica 12.0 StatSoft); significant difference: (*) *p* < 0.05, (**) *p* < 0.01, (***) *p* < 0.001

In addition, the effect of mTOR inhibitor everolimus on caspase-3 activity and proliferation was studied in the range between 20 nM and 10 μM concentration. As the inhibitor concentration increased, so did caspase-3 activity in both cell lines, reaching the highest value of about 10-fold increase relative to the control (*p* < 0.001) for the highest tested concentration of everolimus - 10 μM (Fig. 4a). The use of a mTOR inhibitor everolimus (10 μM) in combination with MEK one – AS-703026 resulted in approximately 25-fold increase of caspase-3 activity (*p* < 0.001). Slightly lower, 15-fold effect (*p* < 0.001), was observed for 20 nM concentration of everolimus (Fig. 4b).

In contrast, cell proliferation decreased with increasing everolimus concentration regardless of the mode of its use: individual or in combinations. 10 μM everolimus combined with the MK-2206 inhibitor led to a decrease of proliferation (about 55%), and to a comparable effect for the combination with MEK inhibitor - AS-703026 (Fig. 4b).

### DNA fragmentation ELISA assay - detection of apoptosis

**Fig. 5:**
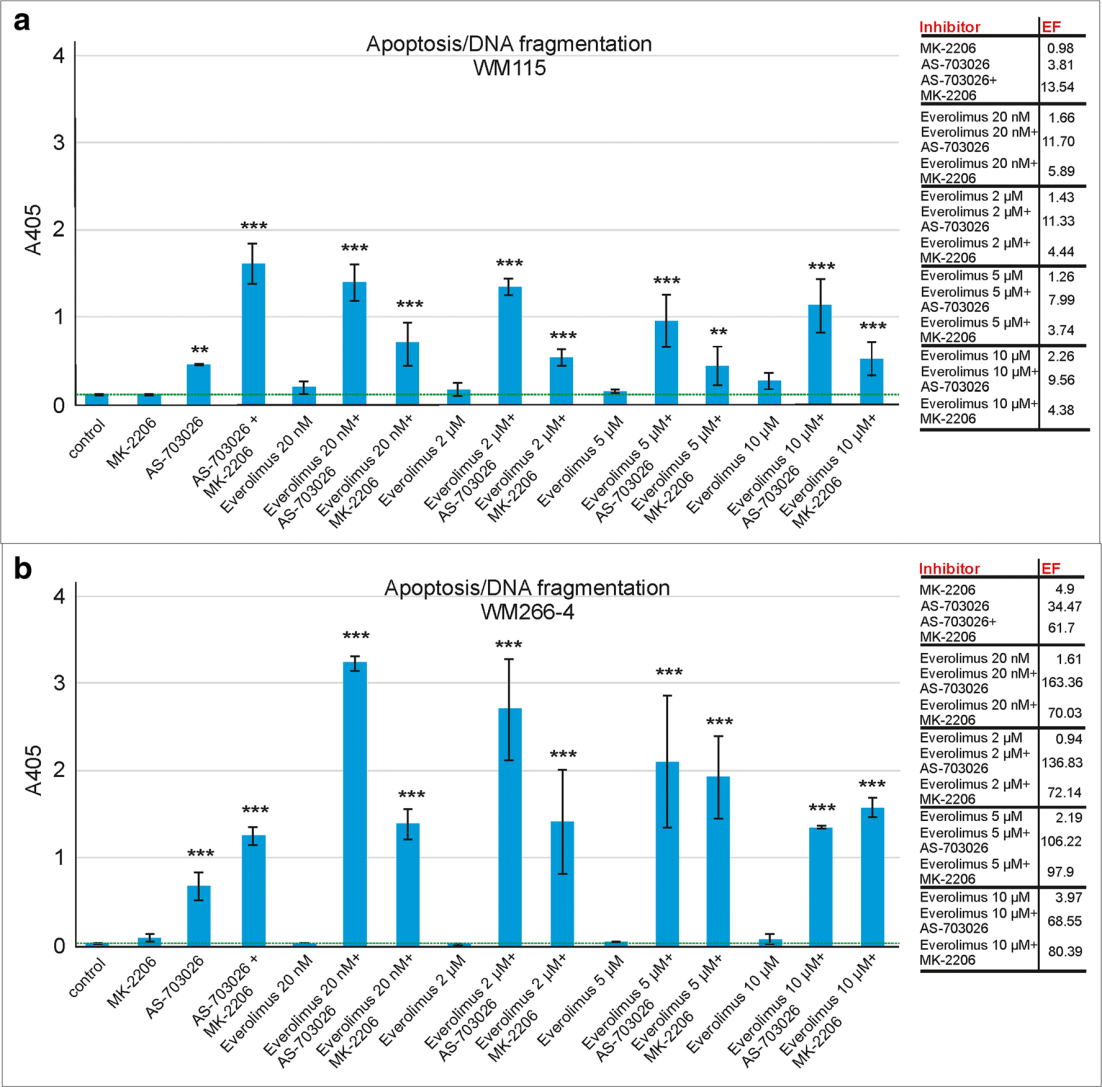
The effect of protein kinase inhibitors on melanoma cell apoptosis - WM115 (**a**) and WM266–4 (**b**). Apoptosis was detected as photometric enzyme-immunoassay for the qualitative and quantitative in vitro determination of cytoplasmic histone-associated-DNA-fragments (mono- and oligonucleosomes). Effect of protein kinase inhibitors and their combinations on apoptosis induction evaluated by the mean of DNA fragmentation ELISA assay after 24 h treatment of melanoma cells. Error bars represent SD. The enrichment factor (EF) was calculated to estimate the fold increase of DNA fragmentation in treated samples with reference to control one. The graphs present mean values of representative experiments performed in duplicates. Statistical analyses were performed using one-way ANOVA with post-hoc Dunett test (Statistica 12.0 StatSoft); significant difference: (*) *p* < 0.05, (**) *p* < 0.01, (***) *p* < 0.001

In order to verify apoptosis induction in the selected samples that were assayed for caspase-3 activity, the cell death detection ELISA assay which reflects DNA fragmentation in apoptotic cells was performed. As demonstrated in Fig. 5a, in the case of WM115 melanoma cells, none of the inhibitors used individually was considerably effective in apoptosis triggering except MEK inhibitor AS-703026, which led to about 4-fold (*p* < 0.01) enrichment in fragmented DNA content.

The highest level of DNA apoptotic degradation was observed in response to concurrent application of MEK inhibitor - AS-703026 with AKT kinase inhibitor – MK-2206 as well as mTOR inhibitor everolimus with MEK inhibitor- AS-703026 (Fig. 5a). It manifested by significant increase in absorbance value as compared to an untreated sample, with the enrichment factor (EF; calculated to estimate the fold increase of DNA fragmentation in treated samples with reference to control one) reaching 13,5 (*p* < 0.001); 11,7 (*p* < 0.001) and 11,3 (*p* < 0.001) for MEK inhibitor – AS-703026 combined with AKT kinase inhibitor – MK-2206, 20 nM everolimus combined with MEK inhibitor – AS-703026 and 2 μM everolimus combined with MEK inhibitor – AS-703026, respectively. The data seem to suggest a synergistic effect of the applied agents. Unexpectedly, further increase of everolimus concentration in combination with AS-703026, both to 5 μ M as well as to 10 μM was not followed by enhancement of apoptosis, showing even lower EF (8,0 (*p* < 0.001) and 9, 6 (*p* < 0.001) respectively).

Combination of mTOR inhibitor everolimus with AKT kinase inhibitor – MK-2206 was about 2-fold, less effective in comparison to everolimus and MEK inhibitor - AS-703026. Independently of everolimus concentration (2, 5, 10 μM), the EF value oscillated around 4. An exception was the combination with the lowest everolimus concentration (20 nM), in which case the EF value reached 5,9 (*p* < 0.001).

Similarly, in the case of WM266–4 melanoma cells (Fig. 5b), each of the inhibitors used individually was hardly effective in apoptosis induction, with the exception of MEK inhibitor – AS-703026, as manifested by high EF value (~35, *p* < 0.001). Nevertheless, combining MEK inhibitor – AS-703026 with AKT kinase inhibitor – MK-2206 did not result in such a significant enhancement as observed in WM115 cells line (Fig. 5b).

The most potent apoptosis induction was observed following concurrent application of 20 nM everolimus with MEK inhibitor – AS-703026, the EF of which equalled 163,3 (*p* < 0.001), which strongly suggests their synergistic cooperation (Fig. 5b). Surprisingly, a further increase of everolimus concentration (2–10 μM) led rather to a considerable decrease of DNA fragmentation in those cells. Still, the EF values were remarkably high (136.8–68,5; *p* < 0.001). The effectiveness of all combinations with AKT kinase inhibitor – MK-2206 was similar, independently of the type and concentration of the accompanying inhibitor and was manifested by high EF value in the range of 70–97 (*p* < 0.001) (Fig. 5b). Besides MEK inhibitor – AS-703026 and AKT kinase inhibitor – MK-2206, all the other combinations seem to indicate synergistic effects on apoptosis induction in WM266–4 melanoma cells (Fig. 5b).

## Discussion

Activation of PI3K/AKT and mTOR pathways is the main development mechanism in many types of cancer, therefore their inhibitors are a promising target in the search for new treatment strategies (Woo et al. 2017). However, the modest antitumour effect of single-agent therapies suggests a need for drug combinations which could reduce their concentrations without the loss of activity in the pursuit of superior clinical responses.

Many literature reports (Ji et al. 2017; Woo et al. 2017) confirm the antitumour efficacy of mTOR inhibitor – rapamycin or another rapalog in combination with highly selective AKT inhibitor – MK2206 in inhibition of proliferation, tumour growth and induction of apoptosis in many types of cancers such as: lymphoblastic leukemia, cholangiocarcinoma, hepatocellular carcinoma, neuroblastoma, gastric cancer, and thyroid cancer.

Our previous studies (Ciołczyk-Wierzbicka and Laidler 2018; Ciołczyk-Wierzbicka et al. 2018) regarding the treatment of melanoma cells with mTOR inhibitors, found that both rapamycin and everolimus had a significant impact on cell cycle regulation, cell proliferation, and invasive potential.

We recently demonstrated that treatment of various melanoma cells with low concentration (5 nM) of mTOR inhibitor everolimus resulted in significantly reduced cell proliferation and diminished number of cancer cells (Ciołczyk-Wierzbicka et al. 2018). It also led to the reduction of metalloproteinase’s (MMPs) activity, as well as reduced invasion, especially when everolimus was used in combination with PI3K/AKT or MEK inhibitors (Ciołczyk-Wierzbicka and Laidler 2018).

The promising preliminary results regarding the use of the mTOR inhibitor everolimus led to the continuation of research on the effects of this agent on pro-survival Bcl-2 family proteins expression. Caspase-3 activity, proliferation, and induction of apoptosis in melanoma cells.

### Expression of pro-survival Bcl-2 family proteins, caspase-3 activity and reduction of cell proliferation

Resistance to apoptosis is an important hallmark of melanoma (Grazia et al. 2014). Enhanced apoptotic cell death is manifested, among others, by: activation of caspase-3, activation pro-apoptotic proteins, and down-regulation of pro-survival protein such as of Bcl-2 and Mcl-1 (Grazia et al. 2014).

Observed was a significant decrease in the expression of studied pro-survival Bcl-2 family proteins: p-Bcl-2 (Ser70), p-Bcl-2 (Thr56), Bcl-2, Bcl-xl, and Mcl-1 after the use of protein kinase inhibitors, which is particularly meaningful when using their combinations. A slight increase in the expression of Bcl-2 and Bcl-2 phosphorylated at serine 70 in the WM266–4 cells after simultaneous blocking of the kinase mTOR pathway (20 nM everolimus) and AKT kinase (MK-2206) may indicate the emergence of drug resistance mechanisms. Observations based on acute myeloid leukaemia [Konopleva et al. 2006] suggest that Mcl-1 expression and basal Bcl-2 phosphorylation may contribute to the drug resistance. During our studies, a decrease in Mcl-1 protein was observed, especially after inhibition of the MEK kinase inhibitor AS-703026, both alone and together with the mTOR - everolimus inhibitor.

In the case of single protein kinase inhibitors, a significant increase in caspase-3 activity was observed after the use of the MEK kinase inhibitor AS-703026; slightly less profound yet similar effect occurred for the ERK1/2 inhibitor - U126. The increase of caspase-3 activity was also observed for the AKT – MK-2206 and PI3K - LY294002 inhibitors.

According to Kim et al. (2010), inhibition of proliferation induced by AS-703026 was mediated by G0-G1 cell cycle arrest and was accompanied by induced apoptosis via caspase-3 and Poly ADP ribose polymerase (PARP) cleavage in multiple myeloma cells, both in the presence and absence of bone marrow stromal cells. Results obtained by Park et al. (2013) suggest that the treatment of malignant melanoma cells (harbouring mutation V600E and resistance to B-RAF inhibitors) with a combination of B-RAF – PLX4032 and MEK – AS-703026 inhibitors significantly induced apoptosis and caspase-3 expression. The use of each of these agents alone was not marked with high efficiency (Park et al. 2013).

Here, the use of combinations of inhibitors gave significantly better results than the use of single inhibitors in terms of suppressing the expression of pro-survival proteins, increasing activity of caspase-3, and decreasing cell proliferation. The observed increase in caspase-3 activity was significantly higher after 48 h treatment, except for the use of the combination of PI3K/AKT and MEK inhibitors in WM266–4 cell line, for which this combination of inhibitors led to elevated LDH activity. In this cell line, observed was a higher increase in caspase-3 activity in a shorter period (24 h), which indicates a faster activation of caspase-3 in this cell line. All this could suggest that the combination of PI3K/AKT and MEK inhibitors caused lysis of WM266–4 cells in addition to their apoptosis.

The treatment of melanoma cells with mTOR inhibitor everolimus (20 nM to 10 μM concentrations) gave promising results, similar to those regarding the effect of this inhibitor on the cell cycle, proliferation, and invasiveness (Ciołczyk-Wierzbicka and Laidler 2018; Ciołczyk-Wierzbicka et al. 2018). The low concentration of the mTOR inhibitor everolimus (20 nM) led to a significant decrease in the expression of all pro-survival Bcl-2 family proteins.

The results obtained by Du et al. (2018) show that everolimus treatment decreased expression of Bcl-2 gene in breast cancer cells.

The use of a combination of mTOR inhibitor – rapamycin and AKT inhibitor – MK-2206 has been described by many research groups that reported a reduction of cell growth, blockade of cell cycle progression, and enhanced apoptosis in different melanoma models (humans, murine and canine) (Grazia et al. 2014).

Numerous data show decreased cell proliferation and increased caspase-3 activity after the use of mTOR inhibitors in many cases of tumours such as pancreatic (Peng and Dou 2017), breast (Woo et al. 2017), and colon cancer (He et al. 2016).

Here, everolimus concentration was found to remain in inverse proportion to proliferation of melanoma cells – the higher the former, the lower the latter. The combination of 10 μM concentration of everolimus with the MK-2206 inhibitor resulted in the decrease in proliferation by 55%. A slightly lower decrease was observed for the combination with AS-703026 inhibitor.

### Apoptosis

Based on the results obtained for the inhibition of pro-survival proteins and caspase-3 activity, the most effective protein kinases inhibitors: AS-703026, MK-2206 and various concentrations (20 nM to 10 μM) of mTOR inhibitor everolimus were selected with the aim of studying the apoptosis activation in melanoma cells.

Among the single inhibitors used, the MEK inhibitor – AS-703026 was the most effective in induction of apoptosis as measured by caspase-3 activation. Similar results were obtained for patients with multiple myeloma (Kim et al. 2010; Kim et al. 2017). Results of the study based on human melanoma cell line with B-RAF V600E mutation that are resistant to B-RAF inhibitors confirmed the efficacy of the AS-703026 inhibitor in the induction of apoptosis and its particular efficacy in combination with the B-RAF inhibitor - PLX4032 (Park et al. 2013).

Herein demonstrated is the fact that everolimus in 10 μM concentration was even more effective than the AKT inhibitor – MK-2206 for the primary melanoma cell line – WM115, while low concentrations of the everolimus induced apoptosis in melanoma cells at a very low level. Everolimus (and another rapalogs) induces apoptosis in a variety of tumours cells: pancreatic cancer (Peng and Dou 2017), ovarian cancer (Guo et al. 2016), colon cancer (He et al. 2016), breast cancer (Du et al. 2018), N-RAS mutant neuroblastoma cell lines (Kiessling et al. 2016), and T cell leukemia/lymphoma in long-term treatment (Darwiche et al. 2011), however in some tumours its high concentration stimulates a caspase-independent pathway – autophagy cell death (Nikoletopoulou et al. 2013; Lui et al. 2016). Autophagy is the process related to cancer progression (Mowers et al. 2017). High level of autophagy can be observed in some rapidly growing cells (Neufeld 2012) and in cells losing cellular adhesion (Dower et al. 2018). Autophagy may also sensitize tumour cells to anticancer drugs (Paquette et al. 2018). The effect of treatment of WM266–4 cells with 10 μM everolimus suggests that high concentration of this rapalog, similarly to PI3K kinase inhibitors and other activators (Milinkovic et al. 2018), might have induced autophagy in the studied metastatic cells (WM266–4).

It is noteworthy that the very low concentrations of the everolimus inhibitor (20 nM) in combination with the MEK inhibitor - AS-703026 led to very significant apoptotic effects. The results presented by Zeng et al. (2018) show that the combination of everolimus and AZD6244 – a highly selective ERK inhibitor, significantly enhanced apoptosis and impaired the viability of renal carcinoma cells, while high concentrations of everolimus alone induce autophagy in these cells. Kiessling et al. (2016) showed that mTOR inhibitor everolimus in single mode use reduces cell growth and leads to apoptosis in N-RAS mutant neuroblastoma cell lines, and in combination with MEK inhibitor produced a synergistic effect and could be important in future clinical studies.

The results obtained by He et al. (2016) regarding the use of high concentrations of everolimus (10-25 μM) in colon cancer cells with the BRAF V600E and K-RAS mutation showed that they are unlikely to respond to monotherapy targeting mTOR inhibitor, but might benefit from combination therapy with PI3K, RAF or MEK inhibitors.

Results of studies from many centres on a panel of human cancer cell lines of different histological origin confirmed the role of PTEN status in determining pharmacological interactions between RAF/MEK and PI3K/AKT/mTOR pathways inhibitors (Weeber et al. 2017; Sathe et al. 2018; Milella et al. 2017). PTEN-loss allowed effective prediction of synergistic inhibitory growth interactions between RAF/MEK and PI3K/AKT/mTOR inhibitors (Milella et al. 2017).

A similar, but lower effect was also observed here for the combination of AKT inhibitor – MK-2206 and everolimus. This combination was more effective in inhibiting melanoma cell proliferation. Our recent study showed that this combination of protein kinase inhibitors had a very promising effect on inhibiting the invasiveness and activity of metalloproteinase 2 and 9 (Ciołczyk-Wierzbicka and Laidler 2018).

MK-2206, a potent oral allosteric AKT inhibitor that enhances the antitumour potency of chemotherapeutic agents (Ji et al. 2017), had no effect on normal peripheral blood mononuclear cells, but induced G1-phase arrest and apoptosis in leukemia cells (Lu et al. 2015), and induction of apoptosis by combined treatment of bufalin in multiple myeloma cells (Xiang et al. 2017).

The herein reported nanomolar concentration of mTOR inhibitor everolimus in the combination with inhibitor of MAP kinase (AS-703026) or AKT kinase (MK-2206) pathway are sufficient to induce apoptosis, and reduce proliferation and invasiveness in melanoma cells. The result seems very important and promising on account of the low concentrations of inhibitors. One can hope that such an approach will find recognition and application in the near future.
